# Fatalism and Interest in Cancer Screening Among African American Individuals

**DOI:** 10.1001/jamanetworkopen.2025.26612

**Published:** 2025-08-13

**Authors:** McKenzie T. Reese, Timothy J. Williamson

**Affiliations:** 1 Department of Psychological Science, Loyola Marymount University, Los Angeles, California

## Abstract

This survey study analyzes a national dataset to assess associations between cancer mortality salience and cancer screening interest among African American individuals.

## Introduction

African American individuals experience disproportionately high cancer mortality compared to White individuals,^1^ partly due to reduced access to high-quality care, including at early stages of the care continuum (eg, screening).^1^ These structural barriers may be perceived as beyond individuals’ control and contribute to fatalistic beliefs about cancer, shaping attitudes toward screening. Cancer fatalism is a multidimensional construct that includes cancer mortality salience (associating cancer with death) and occurrence fatalism (perceiving cancer risk as uncontrollable).^2,3^ Evidence linking fatalism and screening is mixed,^3,4,5^ suggesting a need to analyze its distinct components separately.^3^ Therefore, we used a national dataset to assess how cancer mortality salience and occurrence fatalism are associated with cancer screening interest among African American individuals at a population level in the US.

## Methods

This survey study analyzed data from the 2022 Health Information National Trends Survey (HINTS 6)—a nationally representative survey of US adults (response rate: 28.1%) using a 2-stage stratified sampling design. Surveys were administered via mail and online. Our analytic sample included African American respondents (identified via self-report) without a prior cancer diagnosis. The outcome was interest in cancer screening, measured on a 4-point Likert scale answering the question “How interested are you in having a cancer screening test this year?” from “not at all” to “very.” Independent variables were cancer mortality salience (“When I think about cancer, I automatically think about death.”) and cancer occurrence fatalism (“There’s not much you can do to lower your chances of getting cancer.”), both rated on 4-point scales—from strongly disagree to strongly agree—and reverse scored so higher scores reflected higher fatalism.

We conducted a multivariable linear regression using jackknife replicate weights to obtain accurate SEs for the regression model. The final sample weight was used for population-level point estimates. Covariates included age, gender, and education. Coefficients were considered statistically significant if the 95% CI did not include 0. Participants with information on all study variables were included in analyses, with HINTS-designated missing values recoded as system missing. Analyses were conducted using STATA Version 18. This study was reviewed and designated as exempt research under 45 CFR §46.104 by the Westat institutional review board and the Loyola Marymount University institutional review board. Informed consent was obtained from all individual participants included in the study, which follows STROBE reporting guidelines.

The sample included 613 African American adults without a history of cancer, representing 18.6 million individuals nationally. Weighted estimates indicated that 56.1% were female, 54.3% were ages 18 to 49 years, and 49.3% completed some college. Interest in cancer screening varied (mean, 2.58; 95% CI, 2.40-2.75), with 30.6% “very interested,” 26.0% “somewhat interested,” 13.9% “a little interested,” and 29.5% “not at all interested.”

Regression results (Table) showed that higher cancer mortality salience and female gender were significantly associated with higher screening interest (Figure). Cancer occurrence fatalism, age, and education were not significantly associated with screening interest.

**Table.** Linear Regression Estimating Interest in Cancer Screening Among African American Adults in the US in 2022 (n = 613, Representing 18.60 million individuals)

Variable	b (95% CI)
Cancer mortality salience^a^^,^^b^	0.21 (0.01 to 0.41)^c^
Cancer occurrence fatalism^a^^,^^d^	0.02 (−0.17 to 0.21)
Age (reference group, 18-49 y)	
50-64 y	0.12 (−0.27 to 0.51)
65-74 y	0.28 (−0.10 to 0.66)
≥75 years	−0.11 (−0.73 to 0.50)
Gender (reference group, male)	
Female	0.45 (0.06 to 0.84)^c^
Education (reference group, <HS)	
HS graduate	−0.27 (−0.97 to 0.42)
Some college	−0.11 (−0.75 to 0.53)
College graduate or higher	−0.09 (−0.62 to 0.45)

^a^ Cancer mortality salience and cancer occurrence fatalism were measured on a 4-point Likert scale and reverse scored such that higher scores indicate higher levels of cancer mortality salience and cancer occurrence fatalism, respectively.

^b^ Cancer mortality salience was moderately endorsed (mean, 2.60; 95% CI, 2.47-2.73), with 20.0% indicating “strongly disagree,” 21.9% “somewhat disagree,” 35.9% “somewhat agree,” and 22.2% “strongly agree.”

^c^ *P* < .05.

^d^ Cancer occurrence fatalism was generally low (mean, 2.11; 95% CI, 2.02-2.21), with 30.5% indicating “strongly disagree,” 35.2% “somewhat disagree,” 26.6% “somewhat agree,” and 7.6% “strongly agree.”

**Figure.**
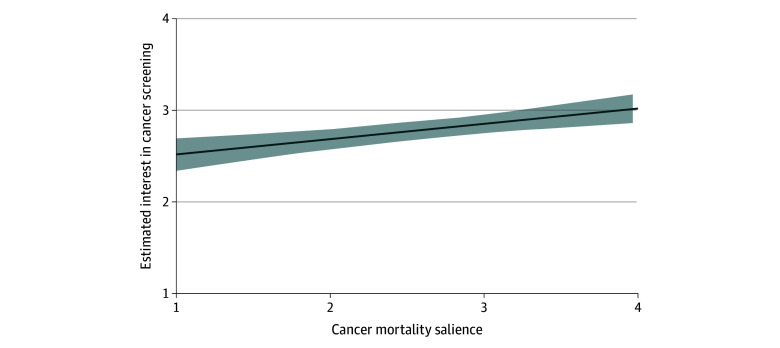
Adjusted Estimates of Interest in Cancer Screening by Cancer Mortality Salience Among African American Health Information National Trends Survey Respondents Higher cancer mortality salience scores were independently associated with higher estimated interest in cancer screening, adjusting for cancer occurrence fatalism, age, gender, and educational attainment. Participants were African American Health Information National Trends Survey respondents who reported no prior history of cancer (n = 613, representing 18.60 million individuals). Shading represents the 95% CI for the line of best fit from the multivariable linear regression model. Cancer mortality salience was measured on a 4-point Likert scale and reverse scored such that higher scores indicate higher levels of cancer mortality salience (“When I think about cancer, I automatically think about death”; 1 = strongly disagree, 2 = somewhat disagree, 3 = somewhat agree, 4 = strongly agree). Cancer screening interest was measured on a 4-point Likert scale (“How interested are you in having a cancer screening test in the next year?”; 1 = not at all, 2 = a little, 3 = somewhat, 4 = very).

## Discussion

This survey study indicated that higher cancer mortality salience—but not occurrence fatalism—among African American adults was associated with greater interest in screening, although the effect size was small. Women reported higher interest than men, consistent with research.^6^ Mortality salience among African American individuals may reflect perceptions of cancer as serious, thus motivating rather than deterring screening interest. The nonsignificant finding for occurrence fatalism may reflect that even those who believe cancer cannot be prevented may still value early detection. Limitations include the cross-sectional design (preventing causal inference), the lack of screening behavior assessment, and that the screening interest item was nonspecific; interest may vary depending on cancer type (eg, mammography, colonoscopy). Future studies should assess the roles of religion and spirituality (not measured in HINTS 6) and explore whether fatalistic beliefs among populations are influenced more by experiences with systemic inequities than by perceptions of cancer outcomes as inherently uncontrollable. Understanding these distinctions could inform culturally sensitive interventions to improve screening rates and reduce racial disparities.

## Notes

Supplement.
Data Sharing Statement
