# Supplementary material for: Fatalism and Interest in Cancer Screening Among African American Individuals
Source: JAMA Netw Open. 2025 Aug 13;8(8):e2526612. doi: 10.1001/jamanetworkopen.2025.26612 (PMC12351406; doi:10.1001/jamanetworkopen.2025.26612)
Supplement: Supplement. — Data Sharing Statement [file jamanetwopen-e2526612-s001.pdf]

Data Sharing Statement

Reese. Fatalism and Interest in Cancer Screening Among African American Individuals. *JAMA Netw Open*. Published August 13, 2025. doi:10.1001/jamanetworkopen.2025.26612

Data
Data available: Yes
Data types: Deidentified participant data
How to access data: https://hints.cancer.gov/data/default.aspx
When available: beginning date: 04-23-2025

Supporting Documents
Document types: Other (please specify)
Additional Information: Methodology Reports
How to access documents: https://hints.cancer.gov/data/methodology-reports.aspx
When available: beginning date: 04-23-2025

Additional Information
Who can access the data: The public
Types of analyses: For any purpose
Mechanisms of data availability: Acknowledgment of terms from NCI HINTS website
